# Reduced occludin expression is related to unfavorable tumor phenotype and poor prognosis in many different tumor types: A tissue microarray study on 16,870 tumors

**DOI:** 10.1371/journal.pone.0321105

**Published:** 2025-04-02

**Authors:** Seyma Büyücek, Florian Viehweger, Viktor Reiswich, Natalia Gorbokon, Viktoria Chirico, Christian Bernreuther, Florian Lutz, Simon Kind, Ria Schlichter, Sören Weidemann, Till S. Clauditz, Andrea Hinsch, Ahmed Abdulwahab Bawahab, Frank Jacobsen, Andreas M. Luebke, David Dum, Claudia Hube-Magg, Martina Kluth, Katharina Möller, Anne Menz, Andreas H. Marx, Till Krech, Patrick Lebok, Christoph Fraune, Guido Sauter, Ronald Simon, Eike Burandt, Sarah Minner, Stefan Steurer, Maximilian Lennartz, Morton Freytag

**Affiliations:** 1 Institute of Pathology, University Medical Center Hamburg-Eppendorf, Hamburg, Germany; 2 Pathology Department, Faculty of Medicine, University of Jeddah, Jeddah, Saudi Arabia; 3 Pathology-Hamburg, Labor Lademannbogen MVZ GmbH, Hamburg, Germany; 4 Department of Pathology, Academic Hospital Fuerth, Fuerth Germany; 5 Institute of Pathology, Clinical Center Osnabrueck, Osnabrueck, Germany; First Affiliated Hospital of Anhui Medical University, CHINA

## Abstract

Occludin is a key component of tight junctions. Reduced occludin expression has been linked to cancer progression in individual tumor types, but a comprehensive and standardized analysis across human tumor types is lacking. To study the prevalence and clinical relevance of occludin expression in cancer, a tissue microarray containing 16,870 samples from 148 different tumor types and 608 samples of 76 different normal tissue types was analyzed by immunohistochemistry. Occludin immunostaining was observed in 10,746 (76.6%) of 14,017 analyzable tumors, including 18.9% with weak, 16.2% with moderate, and 41.6% with strong staining intensity. Occludin positivity was found in 134 of 148 tumor categories and was most frequent in adenocarcinomas (37.5-100%) and neuroendocrine neoplasms (67.9-100%), less common in squamous cell carcinomas (23.8-93%) and in malignant mesotheliomas (up to 48.1%), and rare in Non-Hodgkin’s lymphomas (1-2%) and most mesenchymal tumors. Reduced occludin staining was linked to adverse tumor features in several tumor types, including colorectal adenocarcinoma (advanced pT stage, p < 0.0001; L1 status, p = 0.0384; absence of microsatellite instability, p < 0.0001), pancreatic adenocarcinoma (advanced pT stage, p = 0.005), clear cell renal cell carcinoma (high ISUP grade, p < 0.0001; advanced pT stage, p < 0.0001; high UICC stage, p < 0.0001; distant metastasis, p = 0.0422; shortened overall or recurrence-free survival, p ≤ 0.0116), papillary renal cell carcinoma (high pT stage, p < 0.0001; high UICC stage, p = 0.0228; distant metastasis, p = 0.0338; shortened recurrence-free survival, p = 0.006), and serous high-grade ovarian cancer (advanced pT stage, p = 0.0133). Occludin staining was unrelated to parameters of tumor aggressiveness in breast, gastric, endometrial, and thyroidal cancer. Our data demonstrate significant levels of occludin expression in many different tumor entities and identify reduced occludin expression as a potentially useful prognostic feature in several tumor entities.

## Introduction

Occludin is encoded by the OCLN gene at chromosome 5q13.1 and contributes to the formation of tight junctions between epithelial and endothelial cells [1]. As a member of the tight junction-associated MARVEL domain-containing proteins, occludin interacts with other proteins such as claudins, zonula occludens proteins and junctional adhesion molecules (JAMs) to establish a barrier that controls paracellular permeability [2,3]. Beyond its role in barrier formation, occludin functions as a nicotinamide adenine dinucleotide hydrogen (NADH) oxidase that regulates glucose uptake and ATP production [4] and is also involved in intracellular signaling pathways and cellular processes such as cell proliferation, differentiation, and migration (reviewed in [5]), although the exact mechanisms underlying these functions are still being elucidated.

Occludin is ubiquitously expressed in epithelial cells where it predominantly occurs at cell-cell junctions. Its role is not fully understood. Functional studies have shown that occludin is neither necessary nor sufficient to form tight junctions and to execute pore or barrier functions [6,7]. In experimental cancer cell systems, knockdown of occludin has been linked to decreased epithelial cell-cell adhesion and reduced susceptibility to apoptosis induction in human keratinocytes [8], disoriented epithelial cell migration in Madin-Darby canine kidney (MDCK) cells [9], increased apoptosis and reduced invasion ability of lung cancer cell lines [10], epithelial-mesenchymal transition (EMT) in epithelial cell cultures [11] and increased cell migration and invasion in colon cancer cells [12]. In vivo studies have also shown associations between reduced occludin expression and distant metastasis in breast cancer [13,14], high tumor grade in colon cancer [15,16], lymph node metastasis in endometrial cancer [17], lymph node and peritoneal metastasis in gastric cancer [18], and poor prognosis in esophageal [19] and gall bladder cancer [20]. However, given these functional indications of a tumor-relevant role of reduced occludin expression, the available information on the prevalence and clinical relevance of reduced occludin expression in clinical tumor tissue samples is sparse and partly contradictory. In 36 IHC studies, only 55 different tumor entities have been evaluated. Moreover, in these 3 cancer entities where multiple studies have been executed, the obtained results were considerably discrepant. In these studies, the range of reported occludin positive cases ranged from 37.3 to 100% in colon cancer [21,22], from 52.8 to 98% in serous ovarian cancer [23,24], and from 8.7 to 70.2% in oral squamous cell carcinoma [25,26]. Such conflicting data are typically caused by the use of different antibodies, immunostaining protocols, and criteria to define immunopositivity.

To better understand the prevalence and potential clinical significance of occludin expression in cancer, a comprehensive study analyzing a large number of neoplastic and non-neoplastic tissues under highly standardized conditions is needed. Therefore, occludin expression was analyzed in more than 16,000 tumor tissue samples from 148 different tumor types and subtypes as well as 76 non-neoplastic tissue categories by immunohistochemistry (IHC) in a tissue microarray (TMA) format in this study.

## Materials and methods

### Tissue Microarrays (TMAs)

The normal tissue TMA was composed of 8 samples from 8 different donors for each of 76 different normal tissue types (608 samples on one slide). The cancer TMAs contained a total of 16,870 primary tumors from 148 tumor types and subtypes, distributed across 42 TMA blocks. Each TMA block contains several identical tissues to control the reproducibility of the immunostaining assay. Detailed histopathological data on grade, pathological tumor stage (pT) or pathological lymph node status (pN) were available from 2,351 colon cancers, 1,757 renal cell cancers, 598 pancreatic cancers, and 369 serous ovarian cancers. Clinical follow up data were available from 789 patients with clear cell and from 177 patients with papillary renal cell carcinoma (RCC), with a median follow-up time of 48 and 51 months (range 1-250 and 1-247). Clinical data were assessed for research purposes between 2005 and 2022. The authors of this study had no access to information that could identify individual participants during or after data collection. The composition of both normal and cancer TMAs is described in detail in the results section. All samples were from the archives of the Institutes of Pathology, University Hospital of Hamburg, Germany, the Institute of Pathology, Clinical Center Osnabrueck, Germany, and Department of Pathology, Academic Hospital Fuerth, Germany. Tissues were fixed in 4% buffered formalin and then embedded in paraffin. The TMA manufacturing process was described earlier in detail [27, 28]. In brief, one tissue spot (diameter: 0.6 mm) was transmitted from each cancer containing donor block in an empty recipient paraffin block. The suitability of TMAs made from a single 0.6 mm spot per cancer to find associations between molecular markers and tumor phenotype or patient prognosis has been demonstrated before [29]. The use of anonymized patient data and archived remnants of diagnostic tissues for manufacturing of TMAs and their analysis for research purposes without informed consent has been approved by local laws (HmbKHG, §12) and by the local ethics committee (Ethics commission Hamburg, WF-049/09). All work has been carried out in compliance with the Helsinki Declaration.

### Immunohistochemistry (IHC)

Freshly prepared TMA sections were immunostained on one day in one experiment. Slides were deparaffinized with xylol, rehydrated through a graded alcohol series and exposed to heat-induced antigen retrieval for 5 minutes in an autoclave at 121°C in pH 9 Dako Target Retrieval Solution (Agilent, CA, USA; cat. #S2367). Endogenous peroxidase activity was blocked with Dako Peroxidase Blocking Solution (Agilent, CA, USA; cat. #52023) for 10 minutes. Primary antibody specific against occludin protein (mouse monoclonal, MSVA-415M, MS Validated Antibodies, Hamburg, Germany, cat. #4373-415M-01) was applied at 37°C for 60 minutes at a dilution of 1:150. For validating the specificity of the antibody used in our study, we compared the staining patterns of MSVA-415M with those of a second independent antibody as suggested by the international working group of antibody validation [30]. The normal tissue TMA was also analyzed by the mouse monoclonal occludin antibody OC-310 (Thermo Fisher Scientific, Waltham, MA, USA; cat. #33-1500) at a dilution of 1:25 and an otherwise identical protocol. Bound antibody was then visualized using the EnVision Kit (Agilent, CA, USA; cat. #K5007) according to the manufacturer’s directions. The sections were counterstained with haemalaun. One pathologist (SB) scored all TMA slides. For tumor tissues, the percentage of occludin positive tumor cells was estimated, and the staining intensity was semi-quantitatively recorded (0, 1 + , 2 + , 3+). For statistical analyses, the staining results were categorized into four groups as follows: Negative: no staining at all, weak staining: staining intensity of 1 + in ≤  70% or staining intensity of 2 + in ≤  30% of tumor cells, moderate staining: staining intensity of 1 + in >  70%, staining intensity of 2 + in >  30% but in ≤  70% or staining intensity of 3 + in ≤  30% of tumor cells, strong staining: staining intensity of 2 + in >  70% or staining intensity of 3 + in >  30% of tumor cells. For each tumor, it was recorded whether the staining was only membranous, membranous and cytoplasmic, cytoplasmic and membranous, or cytoplasmic only.

### Statistics

Statistical calculations were performed with JMP 16 software (SAS Institute Inc., NC, USA). Contingency tables and the chi²-test were performed to search for associations between occludin immunostaining and tumor phenotype. For univariate survival analysis, the Log-Rank test was applied to detect significant differences between groups. Survival curves were plotted according to Kaplan-Meier.

## Results

### Technical issues

A total of 14,017 (83%) of 16,870 tumor samples were interpretable in our TMA analysis. Non-interpretable samples demonstrated lack of unequivocal tumor cells or lack of entire tissue spots. A sufficient number of samples (≥4) of each normal tissue type was evaluable.

### Occludin immunostaining in normal tissues

**Fig 1:**
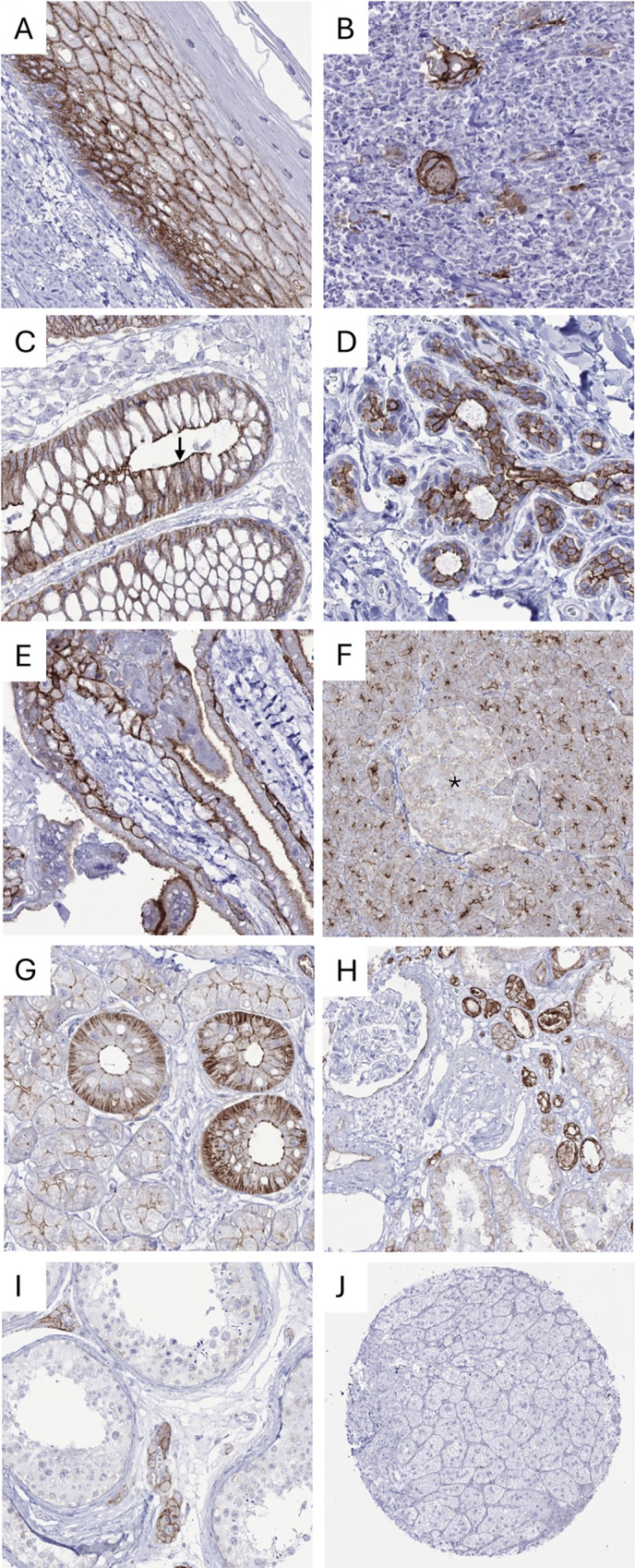
Membranous occludin staining in normal tissues. A) Squamous epithelium of the ectocervix showing intense staining of suprabasal and intermediate cell layers, B) staining in corpuscles of Hassall’s of the thymus, C) membranous staining of the mucosa of the rectum, most intense at the luminal cell membrane (arrow), D) strong staining of luminal cells of the breast glands, E) strong staining of the cytotrophoblast and the apical membrane of the syncytiotrophoblast in the placenta, F) strong staining at apical membranes of acinar cells while there is only a faint staining of islet cells (asterisk, magnification 200x), G) intense staining of excretory ducts and weak staining of glandular cells of the salivary glands, H) kidney with strongest staining in collecting ducts, weaker in distal tubuli, weakest in proximal tubuli and the parietal layer of the Bowman capsule, I) moderate staining of Leydig cells in the testis, J) lack of occludin staining in the adrenal gland.

Occludin immunostaining was predominantly membranous. At variable intensity, it was observed in virtually all epithelial cell types and also in endothelial cells. In squamous epithelium, occludin staining was most intense in suprabasal and intermediate cell layers but less strong or absent in the basal and superficial cell layers. Occludin staining also occurred in corpuscles of Hassall’s of the thymus. In the gastrointestinal tract, gallbladder, endometrium, endocervix, fallopian tube, and respiratory epithelium, a diffuse membranous staining occurred, but staining was most intense at the luminal/apical membranes. In the breast, luminal cells showed a strong staining while basal cells were negative or weakly positive. In the placenta, a moderate to strong membranous staining was seen in the cytotrophoblast and the apical membrane of the syncytiotrophoblast while staining was only weak in amnion cells. In the liver, staining was strong in bile duct cells, but weaker in hepatocytes. In the pancreas, membranous occludin staining was strongest at apical membranes of acinar cells and only weak on islet cells. In salivary glands, occludin staining was most intense in excretory ducts and only faint in glandular cells. In the kidney, occludin staining was strongest in collecting ducts, weaker in distal tubuli and weakest in proximal tubuli as well as in the parietal layer of the Bowman capsule. Occludin staining of epithelial cells was strong in seminal vesicles, follicular cells of the thyroid, moderate in the parathyroid, but only faint in the prostate, the corpus epididymis, and the adenohypophysis. In the testis, staining was limited to Leydig cells. A moderate staining was seen in pneumocytes of the lung. Occludin staining at variable levels was also seen in endothelial cells in all tissues. Representative images are shown in Fig 1. All occludin positive cell types were observed by using both MSVA-415M and OC-3F10 (S1 Fig). Occludin staining did not occur in epithelial cells of the adrenal gland, bone marrow, muscle cells, fat, brain, and the neurohypophysis.

### Occludin immunostaining in neoplastic tissues

**Fig 2:**
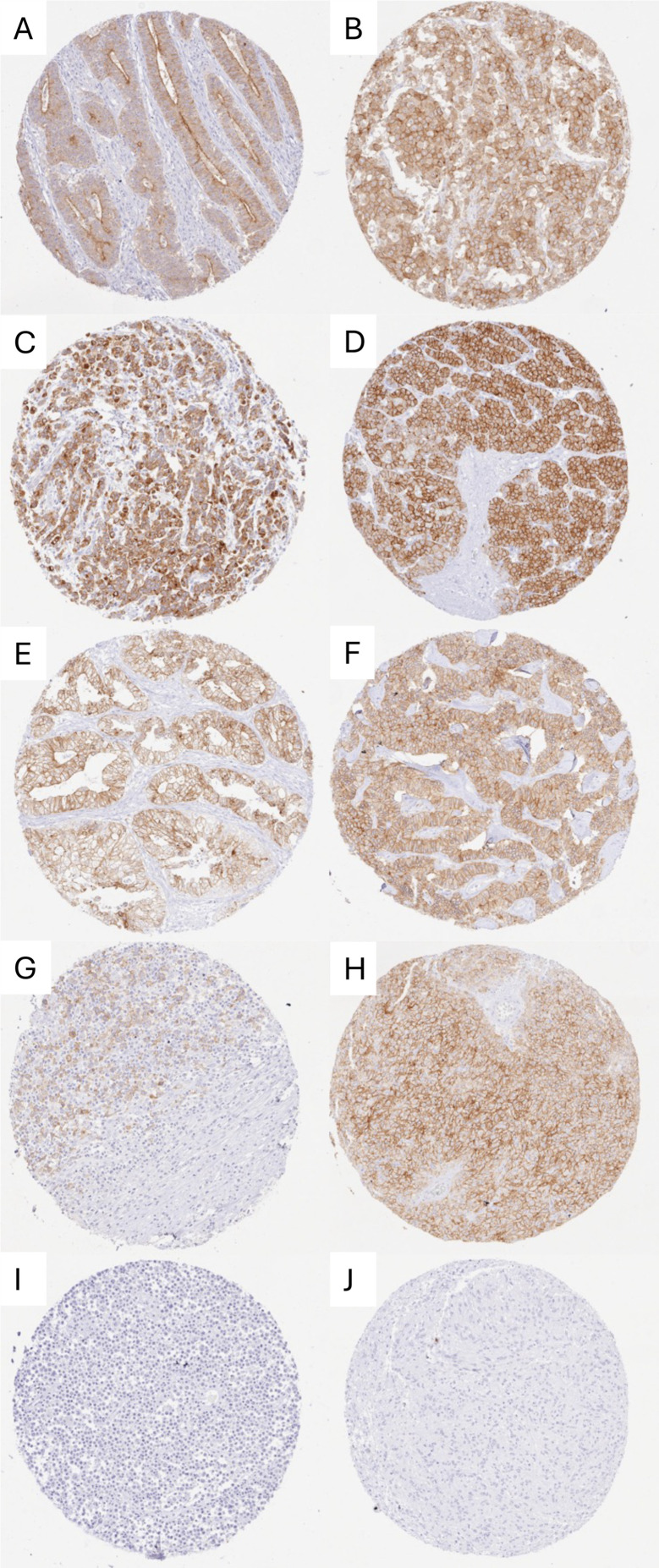
Membranous occludin staining in tumors. A) Prominent staining at the apical membrane of a colorectal adenocarcinoma, B) strong predominantly membranous staining of a breast cancer of no special type, C) adenocarcinoma of the esophagus with strong membranous staining, D) oncocytoma of the kidney with strong membranous positivity, E) clear cell carcinoma of the ovary with moderate membrane staining, F) neuroendocrine tumor of the pancreas with strong membranous positivity, **G)** Hodgkin’s lymphoma with moderate staining of the tumor cells, **H)** Leydig cell tumor of the testis with strong membrane staining, I) occludin-negative diffuse large B-cell lymphoma, J) occludin-negative gastrointestinal stroma tumor.

**Fig 3:**
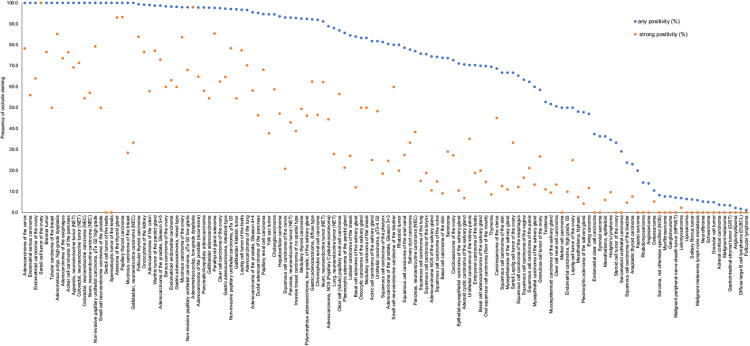
Ranking order of human tumors according to the rate of occludin positivity in human tumors. Both the frequency of positive cases (blue dots) and the frequency of strongly positive cases (orange dots) are shown.

**Fig 4:**
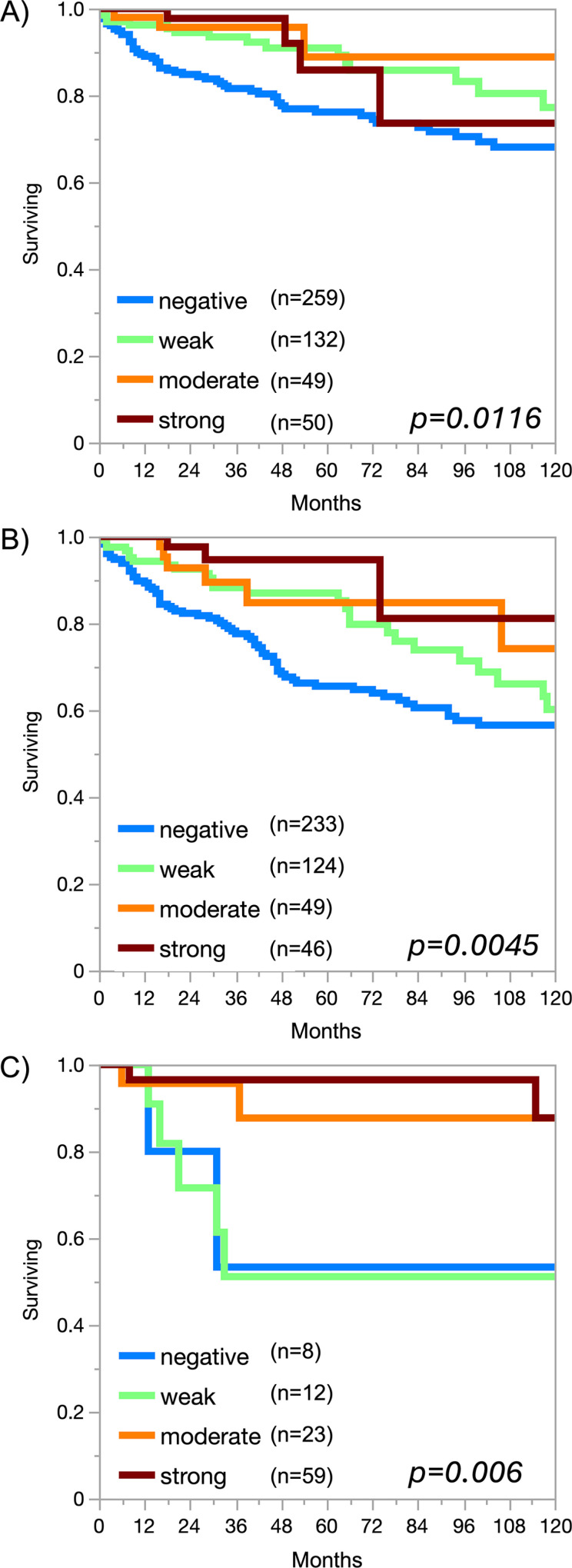
Prognostic relevance of occludin in kidney cancer. Occludin expression and A) overall survival in clear cell carcinomas, B) recurrence-free survival in clear cell carcinoma, and C) recurrence-free survival in papillary carcinomas.

A significant occludin immunostaining was observed in 10,746 (76.7%) of 14,017 analyzable tumors, including 18.9% with weak, 16.2% with moderate, and 41.6% with strong staining intensity. Occludin staining varied both in intensity and in its pattern between samples. Most occludin positive tumors showed a membranous or predominantly membranous staining pattern but some tumors exhibited a purely or predominantly cytoplasmic staining. Representative images are shown in Fig 2. At least an occasional weak occludin positivity was detected in 134 of 148 (90.5%) tumor types and tumor subtypes and 108 (72.9%) entities included at least one case with strong occludin positivity (Table 1). Occludin positivity was most seen in adenocarcinomas (37.5-100%) and in neuroendocrine neoplasms (67.9-100%), slightly less frequently in squamous cell carcinomas (23.8-93%) and in malignant mesotheliomas (up to 48.1%) and only rarely in non-Hodgkin’s lymphomas (1-2%) and in most mesenchymal tumors. Notably, occludin positivity was seen in 34.7% of 72 Hodgkin’s lymphomas. A graphical representation of a ranking order of occludin positive and strongly positive cancers is given in Fig 3. The relationship between occludin expression and tumor phenotype in different cancer types is summarized in Table 2. In colorectal adenocarcinoma, reduced occludin staining was associated with advanced pT stage (p < 0.0001), L1 status (p = 0.0384), and absence of microsatellite instability (p < 0.0001). The relationship between low occludin staining and advanced stage was retained in MSS tumors (p = 0.0359). In clear cell RCC, low occludin staining was strongly linked to poor ISUP (p < 0.0001), Fuhrman (p < 0.0001), and Thoenes (p < 0.0001) grades, advanced pT stage (p < 0.0001), high UICC stage (p < 0.0001), distant metastasis (p = 0.0422; Table 2), non-diploid DNA status (p = 0.0101), as well as shortened overall (p = 0.0166; Fig 4a) and recurrence-free (p = 0.0045; Fig 4b) survival. In papillary RCC, low occludin staining was associated with high pT (p < 0.0001) and UICC stage (p = 0.0228), distant metastasis (p = 0.0338), and shortened recurrence-free survival (p = 0.006; Figure 4c). In ductal adenocarcinoma of the pancreas (p = 0.005) and in high-grade serous ovarian cancer (p = 0.0133), low occludin staining was associated with advanced pT stage. In a mixed cohort of squamous cell carcinomas from 9 different sites of origin, low occludin expression was associated with absence of HPV infection (p = 0.0001, Table 3). The level of occludin immunostaining was unrelated to parameters of tumor aggressiveness in breast cancers of no special type, gastric, endometrial and thyroidal cancer.

**Table 1:** Occludin immunostaining in tumors. See materials and methods section for definition of the immunostaining levels.

			Occludin immunostaining				
	**Tumor entity**	on TMA (n)	analyzable (n)	negative (%)	weak (%)	moderate (%)	strong (%)
**Tumors of the skin**	Basal cell carcinoma of the skin	89	65	26.2	58.5	6.2	9.2
	Benign nevus	29	23	100.0	0.0	0.0	0.0
	Squamous cell carcinoma of the skin	145	126	33.3	38.9	15.1	12.7
	Malignant melanoma	65	57	96.5	1.8	1.8	0.0
	Malignant melanoma lymph node metastasis	86	84	94.0	3.6	2.4	0.0
	Merkel cell carcinoma	2	2	50.0	0.0	50.0	0.0
**Tumors of the head and neck**	Squamous cell carcinoma of the larynx	109	90	24.4	38.9	17.8	18.9
	Squamous cell carcinoma of the pharynx	60	59	18.6	37.3	25.4	18.6
	Oral squamous cell carcinoma (floor of the mouth)	130	116	30.2	44.8	10.3	14.7
	Pleomorphic adenoma of the parotid gland	50	28	14.3	46.4	17.9	21.4
	Warthin tumor of the parotid gland	104	79	1.3	6.3	15.2	77.2
	Adenocarcinoma, NOS (Papillary Cystadenocarcinoma)	14	9	11.1	44.4	0.0	44.4
	Salivary duct carcinoma	15	9	22.2	33.3	11.1	33.3
	Acinic cell carcinoma of the salivary gland	181	88	18.2	40.9	15.9	25.0
	Adenocarcinoma NOS of the salivary gland	109	47	25.5	46.8	17.0	10.6
	Adenoid cystic carcinoma of the salivary gland	180	58	29.3	50.0	13.8	6.9
	Basal cell adenocarcinoma of the salivary gland	25	20	30.0	45.0	5.0	20.0
	Basal cell adenoma of the salivary gland	101	50	16.0	58.0	14.0	12.0
	Epithelial-myoepithelial carcinoma of the salivary gland	53	38	28.9	44.7	15.8	10.5
	Mucoepidermoid carcinoma of the salivary gland	343	230	48.3	33.0	9.1	9.6
	Myoepithelial carcinoma of the salivary gland	21	15	40.0	40.0	6.7	13.3
	Myoepithelioma of the salivary gland	11	9	33.3	55.6	0.0	11.1
	Oncocytic carcinoma of the salivary gland	12	6	16.7	33.3	0.0	50.0
	Polymorphous adenocarcinoma, low grade, of the salivary gland	41	13	7.7	7.7	38.5	46.2
	Pleomorphic adenoma of the salivary gland	53	23	52.2	30.4	13.0	4.3
**Tumors of the lung, pleura and thymus**	Adenocarcinoma of the lung	196	178	3.4	11.8	14.6	70.2
	Squamous cell carcinoma of the lung	80	66	24.2	43.9	16.7	15.2
	Mesothelioma, epithelioid	40	33	63.6	27.3	3.0	6.1
	Mesothelioma, biphasic	29	27	51.9	29.6	11.1	7.4
	Thymoma	29	23	30.4	39.1	21.7	8.7
	Lung, neuroendocrine tumor (NET)	29	25	12.0	32.0	28.0	28.0
**Tumors of the female genital tract**	Squamous cell carcinoma of the vagina	30	30	36.7	26.7	20.0	16.7
	Squamous cell carcinoma of the vulva	107	101	25.7	46.5	12.9	14.9
	Squamous cell carcinoma of the cervix	88	86	7.0	41.9	30.2	20.9
	Adenocarcinoma of the cervix	23	23	0.0	17.4	4.3	78.3
	Endometrioid endometrial carcinoma	288	236	1.7	11.9	23.3	63.1
	Endometrial serous carcinoma	36	25	0.0	20.0	24.0	56.0
	Carcinosarcoma of the uterus	57	51	31.4	17.6	5.9	45.1
	Endometrial carcinoma, high grade, G3	13	10	50.0	30.0	10.0	10.0
	Endometrial clear cell carcinoma	9	8	62.5	37.5	0.0	0.0
	Endometrioid carcinoma of the ovary	93	75	0.0	10.7	25.3	64.0
	Serous carcinoma of the ovary	530	417	1.7	16.1	22.3	60.0
	Mucinous carcinoma of the ovary	75	49	2.0	6.1	8.2	83.7
	Clear cell carcinoma of the ovary	51	40	2.5	12.5	22.5	62.5
	Carcinosarcoma of the ovary	47	44	27.3	27.3	18.2	27.3
	Granulosa cell tumor of the ovary	44	41	41.5	12.2	19.5	26.8
	Leydig cell tumor of the ovary	4	4	50.0	0.0	25.0	25.0
	Sertoli cell tumor of the ovary	1	1	0.0	0.0	0.0	100.0
	Sertoli Leydig cell tumor of the ovary	3	3	33.3	33.3	0.0	33.3
	Steroid cell tumor of the ovary	3	3	66.7	33.3	0.0	0.0
	Brenner tumor	32	30	0.0	3.3	20.0	76.7
**Tumors of the breast**	Invasive breast carcinoma of no special type	499	458	7.2	28.2	25.8	38.9
	Lobular carcinoma of the breast	150	129	15.5	33.3	24.0	27.1
	Medullary carcinoma of the breast	8	6	16.7	16.7	16.7	50.0
	Tubular carcinoma of the breast	2	2	0.0	0.0	50.0	50.0
	Mucinous carcinoma of the breast	7	7	0.0	14.3	57.1	28.6
**Tumors of the digestive system**	Adenomatous polyp, low-grade dysplasia	50	48	2.1	0.0	0.0	97.9
	Adenomatous polyp, high-grade dysplasia	50	47	0.0	2.1	12.8	85.1
	Adenocarcinoma of the colon	2483	2130	0.9	13.8	27.4	57.8
	Gastric adenocarcinoma, diffuse type	215	165	7.9	17.6	12.1	62.4
	Gastric adenocarcinoma, intestinal type	215	181	2.8	11.6	21.0	64.6
	Gastric adenocarcinoma, mixed type	62	55	1.8	12.7	25.5	60.0
	Adenocarcinoma of the esophagus	83	53	0.0	13.2	13.2	73.6
	Squamous cell carcinoma of the esophagus	76	49	34.7	44.9	8.2	12.2
	Squamous cell carcinoma of the anal canal	91	84	21.4	28.6	22.6	27.4
	Cholangiocarcinoma	121	109	5.5	16.5	19.3	58.7
	Gallbladder adenocarcinoma	51	44	2.3	15.9	27.3	54.5
	Gallbladder Klatskin tumor	42	33	3.0	9.1	33.3	54.5
	Hepatocellular carcinoma	312	297	6.4	24.6	21.9	47.1
	Ductal adenocarcinoma of the pancreas	659	560	4.8	26.3	22.5	46.4
	Pancreatic/Ampullary adenocarcinoma	98	93	2.2	20.4	19.4	58.1
	Acinar cell carcinoma of the pancreas	18	17	0.0	17.6	5.9	76.5
	Gastrointestinal stromal tumor (GIST)	62	59	96.6	3.4	0.0	0.0
	Appendix, neuroendocrine tumor (NET)	25	13	0.0	23.1	7.7	69.2
	Colorectal, neuroendocrine tumor (NET)	12	7	0.0	0.0	28.6	71.4
	Ileum, neuroendocrine tumor (NET)	53	45	8.9	15.6	13.3	62.2
	Pancreas, neuroendocrine tumor (NET)	101	86	7.0	23.3	26.7	43.0
	Colorectal, neuroendocrine carcinoma (NEC)	14	11	0.0	36.4	9.1	54.5
	Ileum, neuroendocrine carcinoma (NEC)	8	7	0.0	14.3	28.6	57.1
	Gallbladder, neuroendocrine carcinoma (NEC)	4	3	0.0	33.3	33.3	33.3
	Pancreas, neuroendocrine carcinoma (NEC)	14	13	23.1	7.7	30.8	38.5
**Tumors of the urinary system**	Non-invasive papillary urothelial carcinoma, pTa G2 low grade	177	144	2.1	9.0	20.8	68.1
	Non-invasive papillary urothelial carcinoma, pTa G2 high grade	141	106	0.0	8.5	12.3	79.2
	Non-invasive papillary urothelial carcinoma, pTa G3	219	102	2.9	4.9	13.7	78.4
	Urothelial carcinoma, pT2-4 G3	735	527	18.2	21.6	11.8	48.4
	Squamous cell carcinoma of the bladder	22	21	76.2	14.3	4.8	4.8
	Small cell neuroendocrine carcinoma of the bladder	5	5	20.0	20.0	0.0	60.0
	Sarcomatoid urothelial carcinoma	25	24	70.8	12.5	16.7	0.0
	Urothelial carcinoma of the kidney pelvis	62	54	29.6	18.5	16.7	35.2
	Clear cell renal cell carcinoma	1287	1189	49.4	27.6	9.7	13.4
	Papillary renal cell carcinoma	368	317	5.4	10.7	15.8	68.1
	Clear cell (tubulo) papillary renal cell carcinoma	26	23	13.0	17.4	13.0	56.5
	Chromophobe renal cell carcinoma	170	148	8.1	21.6	23.6	46.6
	Oncocytoma of the kidney	257	222	0.9	11.7	10.8	76.6
**Tumors of the male genital organs**	Adenocarcinoma of the prostate, Gleason 3 + 3	83	77	19.5	36.4	19.5	24.7
	Adenocarcinoma of the prostate, Gleason 4 + 4	80	67	4.5	13.4	23.9	58.2
	Adenocarcinoma of the prostate, Gleason 5 + 5	85	74	1.4	9.5	16.2	73.0
	Adenocarcinoma of the prostate (recurrence)	258	187	2.1	12.8	20.3	64.7
	Small cell neuroendocrine carcinoma of the prostate	2	2	0.0	50.0	0.0	50.0
	Seminoma	682	544	26.3	30.7	14.0	29.0
	Embryonal carcinoma of the testis	54	37	29.7	32.4	18.9	18.9
	Leydig cell tumor of the testis	31	31	3.2	6.5	12.9	77.4
	Sertoli cell tumor of the testis	2	2	0.0	100.0	0.0	0.0
	Sex cord stromal tumor of the testis	1	1	100.0	0.0	0.0	0.0
	Spermatocytic tumor of the testis	1	1	0.0	100.0	0.0	0.0
	Yolk sac tumor	53	37	5.4	24.3	32.4	37.8
	Teratoma	53	36	47.2	25.0	16.7	11.1
	Squamous cell carcinoma of the penis	92	85	37.6	30.6	10.6	21.2
**Tumors of endocrine organs**	Adenoma of the thyroid gland	113	101	0.0	1.0	5.9	93.1
	Papillary thyroid carcinoma	391	368	0.0	0.3	6.5	93.2
	Follicular thyroid carcinoma	154	142	0.7	2.1	13.4	83.8
	Medullary thyroid carcinoma	111	93	7.5	22.6	20.4	49.5
	Parathyroid gland adenoma	43	41	2.4	9.8	2.4	85.4
	Anaplastic thyroid carcinoma	45	39	76.9	10.3	5.1	7.7
	Adrenal cortical adenoma	48	34	100.0	0.0	0.0	0.0
	Adrenal cortical carcinoma	27	27	96.3	0.0	3.7	0.0
	Pheochromocytoma	51	50	100.0	0.0	0.0	0.0
**Tumors of hematopoetic and lymphoid tissues**	Hodgkin’s lymphoma	103	72	65.3	5.6	19.4	9.7
	Small lymphocytic lymphoma, B-cell type (B-SLL/B-CLL)	50	50	100.0	0.0	0.0	0.0
	Diffuse large B cell lymphoma (DLBCL)	113	113	98.2	0.9	0.9	0.0
	Follicular lymphoma	88	88	98.9	1.1	0.0	0.0
	T-cell non-Hodgkin’s lymphoma	25	25	100.0	0.0	0.0	0.0
	Mantle cell lymphoma	18	18	100.0	0.0	0.0	0.0
	Marginal zone lymphoma	16	16	100.0	0.0	0.0	0.0
	Diffuse large B-cell lymphoma (DLBCL) in the testis	16	16	100.0	0.0	0.0	0.0
	Burkitt lymphoma	5	5	100.0	0.0	0.0	0.0
**Tumors of soft tissue and bone**	Granular cell tumor	23	20	95.0	5.0	0.0	0.0
	Leiomyoma	50	44	100.0	0.0	0.0	0.0
	Leiomyosarcoma	94	87	93.1	4.6	0.0	2.3
	Liposarcoma	96	93	93.5	6.5	0.0	0.0
	Malignant peripheral nerve sheath tumor (MPNST)	15	14	92.9	7.1	0.0	0.0
	Myofibrosarcoma	26	26	92.3	7.7	0.0	0.0
	Angiosarcoma	42	36	86.1	13.9	0.0	0.0
	Angiomyolipoma	91	89	97.8	2.2	0.0	0.0
	Dermatofibrosarcoma protuberans	21	15	100.0	0.0	0.0	0.0
	Ganglioneuroma	14	13	92.3	7.7	0.0	0.0
	Kaposi sarcoma	8	5	80.0	20.0	0.0	0.0
	Neurofibroma	117	114	94.7	5.3	0.0	0.0
	Sarcoma, not otherwise specified (NOS)	74	72	91.7	6.9	1.4	0.0
	Paraganglioma	41	40	100.0	0.0	0.0	0.0
	Ewing sarcoma	23	17	52.9	23.5	11.8	11.8
	Rhabdomyosarcoma	7	7	85.7	14.3	0.0	0.0
	Schwannoma	122	118	94.9	2.5	2.5	0.0
	Synovial sarcoma	12	11	63.6	36.4	0.0	0.0
	Osteosarcoma	19	19	89.5	5.3	0.0	5.3
	Chondrosarcoma	15	11	100.0	0.0	0.0	0.0
	Rhabdoid tumor	5	5	20.0	20.0	40.0	20.0
	Solitary fibrous tumor	17	16	93.8	6.3	0.0	0.0

**Table 2:** Occludin immunostaining and prognosis colorectal adenocarcinomas, clear cell and papillary carcinomas of the kidney, serous ovarian cancers, and pancreatic adenocarcinomas. See materials and methods section for definition of the immunostaining levels.

				Occludin immunostaining				
			n	negative (%)	weak (%)	moderate (%)	strong (%)	P
**Colon adenocarcinoma**	Tumor stage	pT1	75	0	8	16	76	<0.0001
		pT2	393	0	9.4	26.2	64.4	
		pT3	1140	1.1	14.1	29.1	55.7	
		pT4	408	1.2	18.6	29.2	51	
	Nodal stage	pN0	1053	0.8	12.3	27.5	59.4	0.0542
		pN +	956	1	15.9	28.9	54.2	
	Lymph vessel stage	L0	648	0.8	11.7	26.5	61	0.0384
		L1	1338	0.9	15.4	29.1	54.6	
	Tumor localization	left colon	1100	0.6	14.1	27.1	58.2	0.1159
		right colon	410	1.2	10.2	25.9	62.7	
	MMR status	defective	78	0	1.3	15.4	83.3	<0.0001
		proficient	1059	0.5	12	27.4	60.2	
	RAS mutation status	mutated	321	0.3	14.3	24	61.4	0.272
		wildtype	427	1.2	13.1	28.3	57.4	
	BRAF mutation status	mutated	18	0	16.7	11.1	72.2	0.5826
		wildtype	101	1	9.9	21.8	67.3	
**Clear cell renal cell cancers**	ISUP stage	1	264	53.8	26.1	7.6	12.5	<0.0001
		2	403	40.7	31.5	11.7	16.1	
		3	266	54.9	25.6	7.9	11.7	
		4	75	78.7	16	1.3	4	
	Fuhrman grade	1	62	33.9	32.3	9.7	24.2	<0.0001
		2	682	45.3	29.6	10.9	14.2	
		3	296	55.1	24.7	9.1	11.1	
		4	90	77.8	16.7	1.1	4.4	
	Thoenes grade	1	349	49.9	27.2	10	12.9	<0.0001
		2	491	51.3	28.1	10.4	10.2	
		3	100	77	19	1	3	
	UICC stage	1	329	46.8	26.7	10.6	15.8	<0.0001
		2	37	56.8	29.7	2.7	10.8	
		3	91	64.8	23.1	6.6	5.5	
		4	76	72.4	21.1	5.3	1.3	
	pT stage	1	677	42.7	27.9	11.7	17.7	<0.0001
		2	132	56.1	28.8	5.3	9.8	
		3-4	326	62.3	25.8	6.7	5.2	
	pN stage	0	173	57.8	24.3	10.4	7.5	0.1172
		≥1	25	68	20	0	12	
	pM	0	112	53.6	25.9	8	12.5	0.0422
		≥1	94	68.1	21.3	7.4	3.2	
**Papillary cell renal cell cancers**	ISUP stage	1	39	7.7	7.7	20.5	64.1	0.1389
		2	129	3.9	8.5	14	73.6	
		3	80	6.3	15	20	58.8	
		4	5	40	0	0	60	
	Fuhrman grade	1	4	0	0	25	75	0.2646
		2	176	4.5	9.7	14.2	71.6	
		3	81	6.2	11.1	19.8	63	
		4	9	22.2	33.3	11.1	33.3	
	Thoenes grade	1	55	5.5	9.1	12.7	72.7	0.1256
		2	152	5.9	11.2	17.8	65.1	
		3	16	12.5	31.3	25	31.3	
	UICC stage	1	100	4	8	20	68	0.0228
		2	13	15.4	23.1	15.4	46.2	
		3	5	20	20	20	40	
		4	12	8.3	41.7	33.3	16.7	
	pT stage	1	204	2.9	6.9	15.2	75	<0.0001
		2	45	11.1	13.3	24.4	51.1	
		3-4	32	18.8	28.1	9.4	43.8	
	pN stage	0	24	4.2	12.5	20.8	62.5	0.1845
		≥1	15	20	26.7	20	33.3	
	pM	0	24	4.2	8.3	20.8	66.7	0.0338
			12	16.7	41.7	16.7	25	
**Serous ovarian cancer**	Tumor stage	pT1	27	0	7.4	11.1	81.5	0.0133
		pT2	39	0	10.3	20.5	69.2	
		pT3	216	2.3	23.1	24.1	50.5	
	Nodal stage	pN0	65	1.5	15.4	15.4	67.7	0.1229
		pN1	141	2.8	20.6	26.2	50.4	
**Pancreatic adenocarcinoma**	Tumor stage	pT1	11	0	9.1	27.3	63.6	0.005
		pT2	63	1.6	17.5	38.1	42.9	
		pT3	349	5.2	32.7	21.5	40.7	
		pT4	27	14.8	29.6	33.3	22.2	
	Grade	1	15	6.7	33.3	20	40	0.6668
		2	322	4.3	28	25.5	42.2	
		3	92	6.5	35.9	18.5	39.1	
	Nodal stage	pN0	95	9.5	27.4	21.1	42.1	0.1523
		pN +	354	3.7	30.5	25.4	40.4	

**Table 3:** Occludin immunostaining and prognosis in squamous cell carcinomas of various origin. See materials and methods section for definition of the immunostaining levels.

	HPV status	n	Occludin immunostaining (%)				p
			negative	weak	moderate	strong	
All squamous cell cancers	negativ	282	30.1	38.3	17	14.5	<0.0001
	positive	238	14.7	37.4	23.1	24.8	
Oral squamous cell carcinoma	negativ	63	34.9	36.5	14.3	14.3	0.3399
	positive	12	16.7	41.7	8.3	33.3	
Squamous cell carcinoma of the pharynx	negativ	22	31.8	31.8	27.3	9.1	0.043
	positive	34	5.9	41.2	26.5	26.5	
Squamous cell carcinoma of the larynx	negativ	50	24	36	20	20	0.9573
	positive	8	25	37.5	12.5	25	
Squamous cell carcinoma of the cervix	negativ	10	10	50	20	20	0.8458
	positive	67	6	43.3	32.8	17.9	
Squamous cell carcinoma of the vagina	negativ	15	46.7	33.3	6.7	13.3	0.2171
	positive	15	26.7	20	33.3	20	
Squamous cell carcinoma of the vulva	negativ	53	30.2	47.2	13.2	9.4	0.0271
	positive	26	7.7	46.2	15.4	30.8	
Squamous cell carcinoma of the penis	negativ	27	48.1	29.6	7.4	14.8	0.515
	positive	45	33.3	28.9	13.3	24.4	
Squamous cell carcinoma of the skin	negativ	37	16.2	40.5	24.3	18.9	0.3589
	positive	1	0	0	0	100	
Squamous cell carcinoma of the anal canal	negativ	5	20	40	40	0	0.3399
	positive	30	13.3	33.3	23.3	30	

## Discussion

**Fig 5:**
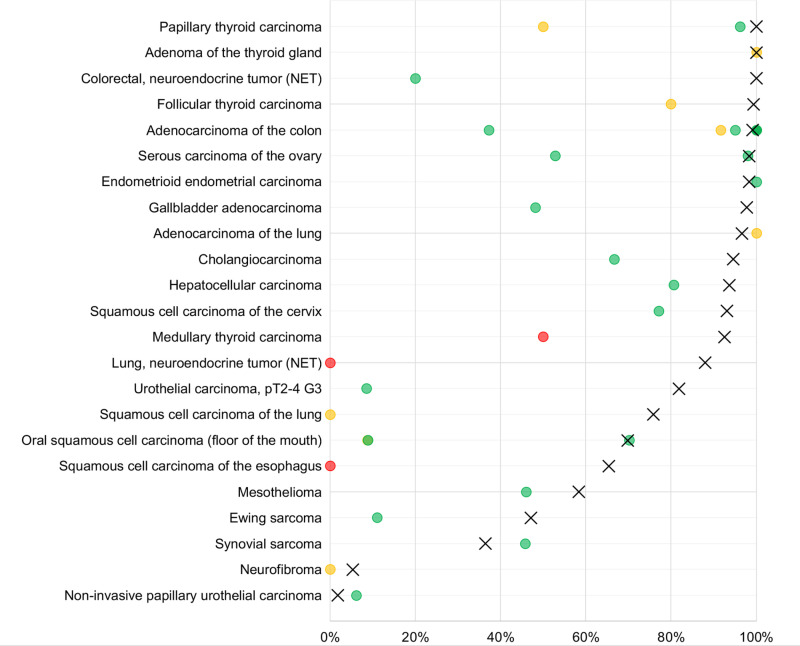
Comparison with previous occludin literature. An „X“ indicates the fraction of occludin positive cancers in the present study, dots indicate the reported frequencies from the literature for comparison: red dots, <  10 analyzed tumors; yellow dots, > 10-25 analyzed tumors; green dots, > 25 analyzed tumors.

The data from our successful analysis of 14,017 tumors from 148 different tumor categories provide a comprehensive overview of occludin expression in cancer. Although occludin expression could be found in almost every tumor entity, occludin positivity was most seen in adenocarcinomas and in neuroendocrine neoplasms. It was slightly less frequently seen in squamous cell carcinomas and in malignant mesotheliomas, and only rarely in non-Hodgkin’s lymphomas and in most mesenchymal tumors. Although previous IHC studies on occludin were limited in number and had provided partly conflicting data (summarized in Fig 5), several earlier studies are in line with our data. For example, 6 of 7 studies analyzing between 12 and 127 colon cancers [15,16,21,31–33] reported occludin positivity rates between 91.6 and 100% (our study: 99.1%). Tobioka et al. [17] found occludin expression in 100% of 42 endometrioid endometrial carcinomas (our study: 98.3%), Dos Santos et al. [24] in 97.5% of 602 serous ovarian carcinomas (our study: 98.2%) and in 46% of 87 mesotheliomas (our study: 58.3%), Montaro et al. [26] in 70.2% of 60 oral squamous cell carcinomas (our study: 69.8%), Billings et al. [34] in 45.7% of 35 synovial sarcomas (our study: 36.4%), and Nakanishi et al. [35] in 6.1% of non-invasive urinary bladder cancers (our study: 1.7%).

That occludin immunostaining of epithelial tumors was often reduced as compared to their cells of origin is in agreement with various earlier reports suggesting a critical role of occludin downregulation for various cancer driving mechanisms such as reduction of cell adhesion and facilitation of cell dissociation, increased motility and proliferation, invasive tumor growth, and epithelial-mesenchymal transition. For example, decreased or disrupted occludin expression has been linked to decreased epithelial cell-cell adhesion, reduced susceptibility to apoptosis induction, and altered Ca(2+)-homeostasis in keratinocytes [8], disorganization of the actin cytoskeleton in epithelial cells [9], accelerated migration and invasion in liver cancer cell lines [36], cell dissociation and activation of the growth signaling pathways in pancreatic cancer cell lines [37], epithelial-mesenchymal transition in cultured mouse epithelial cells [11], and increased tumorigenic and metastatic properties in various cancer cell types [38].

In line with these functional data, our findings revealed a significant link between reduced occludin expression and unfavorable histopathological and clinical tumor parameters in several different cancer entities including colorectal and pancreatic adenocarcinoma, serous high-grade ovarian cancer, as well as clear cell and papillary renal cell carcinoma (RCC). Possible mechanistic interactions are for example known from clear cell RCC, which is characterized by inactivating mutations of the VHL gene. VHL loss of function leads to an accumulation of the hypoxia-inducible factors HIF-1α and HIF-2α, which in turn suppress the expression of tight junction proteins including occludin [39]. It is of note that a tendency towards unfavorable phenotype was also seen for additional tumor entities in this study, although the statistical level of significance was not reached. A relationship between reduced occludin expression and unfavorable tumor phenotype or poor prognosis has earlier been reported for carcinomas of the breast [14], gallbladder [20], gastrointestinal tract [15,18], endometrial cancer [17] as well as squamous cell carcinomas of the esophagus [19]. Another study described significantly lower levels of occludin in metastasis than in primary hepatocellular carcinoma [40]. Based on all these findings, it appears that reduced occludin expression may constitute a critical feature of ominous prognosis in various tumor entities that could be clinically useful. This might for example apply for RCC where high-risk patients are increasingly selected for adjuvant therapy [41].

It is of note that de-novo expression or upregulation of occludin was also found in cancers that are derived from occludin negative cell types. For example, unequivocal occludin staining was identified in 34.7% (9.7% strong) of Hodgkin’s lymphomas, 47.1% (18.8%) of Ewing sarcomas, 6.9% (2.3%) of leiomyosarcomas, and in 10.5% (5.3%) of osteosarcomas. While the number of analyzed tumors of these categories was too small to assess the potential clinical significance of elevated occludin expression, it appears possible that other functionalities than the rather tumor-suppressive tight-junction role of occludin might become effective in these tumors and perhaps provide a growth or survival benefit to affected tumor cells. Few data from earlier studies have indeed highlighted potential tumor promoting effects of elevated occludin expression such as promotion of glucose uptake [4], increased angiogenesis [42], modulation of growths signaling pathways such as MAPK/ERK [43,44], PI3K/Akt [43], and the Hippo pathway [45], or increased tumor aggressiveness after occludin knockdown in lung cancer cells [10]. Earlier studies confirmed occludin protein expression in small subsets of Ewing sarcomas [46] and reported occludin mRNA expression from osteoblasts and osteosarcoma cell lines [47]. The authors of these studies concluded from their findings, however, that occludin may not exert a direct tumor promoting role in theses tumors. Schütz et al. [46] observed occasional formation of tight junction-like structures in Ewing sarcomas, but these structures always lacked at least one of the relevant tight junction proteins (such as occludin, desmins, desmogleins or desmoplakins) and were considered non-functional. Jian et al. [47] compared occludin expression in primary and metastatic osteosarcoma cell lines but did not find obvious differences. It can, thus, not be excluded that the rare expression of occludin in these tumors just reflects random gene expression deregulation in cancer cells that undergo progressing dedifferentiation. It is well known that most deregulated genes in cancer are not relevant for tumor cell behavior [48,49].

In the light of the large scale of our study, we extensively validated the specificity of the occludin antibody. The international working group of antibody validation recommends validating antibodies for the use in immunohistochemistry by either comparing the staining results with a different method for expression analysis or with the staining results obtained by an independent second antibody [30]. As a comparison with a method using extracted RNAs or proteins is impractical because of the ubiquitous presence of occludin positive endothelial and epithelial cells in virtually all organs [50–53], our validation was limited to an antibody comparison. To ensure an as broad as possible range of proteins to be tested for a possible cross-reactivity, 76 different normal tissue categories were included in this analysis. These diverse tissues are likely to contain a large proportion of the proteins expressed in cells of adult humans. Validity of our IHC assay was supported by the confirmation of all cell types found to be occludin positive and of all the characteristic variations of staining intensities such as the conspicuous differences between specific layers of squamous epithelium by the validation antibody OC-3F10.

## Conclusion

Our data demonstrate significant levels of occludin expression in many different tumor entities and identify loss of occludin expression as a potentially useful prognostic marker in several tumor entities.

## Supporting information

S1 Fig IHC validation by comparison of two independent occludin antibodies. Using MSVA-415M, membranous staining was seen in the suprabasal and intermediate cell layers of squamous epithelium of the ectocervix (A), cytotrophoblast and the apical membrane of the syncytiotrophoblast in the placenta (B), hepatocytes of the liver (C), Leydig cells of the testis (D), and apical membranes of acinar cells of the pancreas (E). Using clone OC-3F10, a comparable, although slightly weaker, staining was seen in the ectocervix (a), the placenta (b), the liver (c), the testis (d), and the pancreas (e). The images A-E and a-e are from consecutive tissue sections.
(PDF)
