# Supplementary figures and images for: Reduced occludin expression is related to unfavorable tumor phenotype and poor prognosis in many different tumor types: A tissue microarray study on 16,870 tumors
Source: PLoS One. 2025 Apr 2;20(4):e0321105. doi: 10.1371/journal.pone.0321105 (PMC11964279; doi:10.1371/journal.pone.0321105)

MSVA-415M

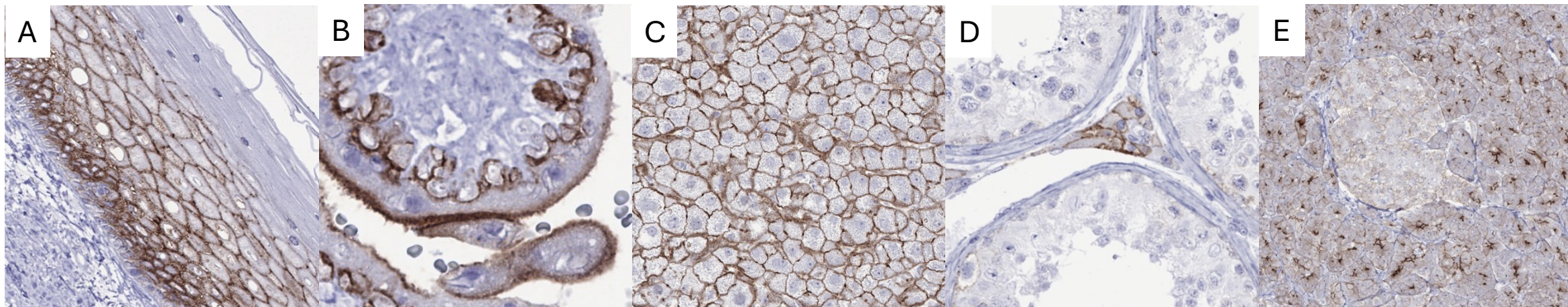

OC-3F10

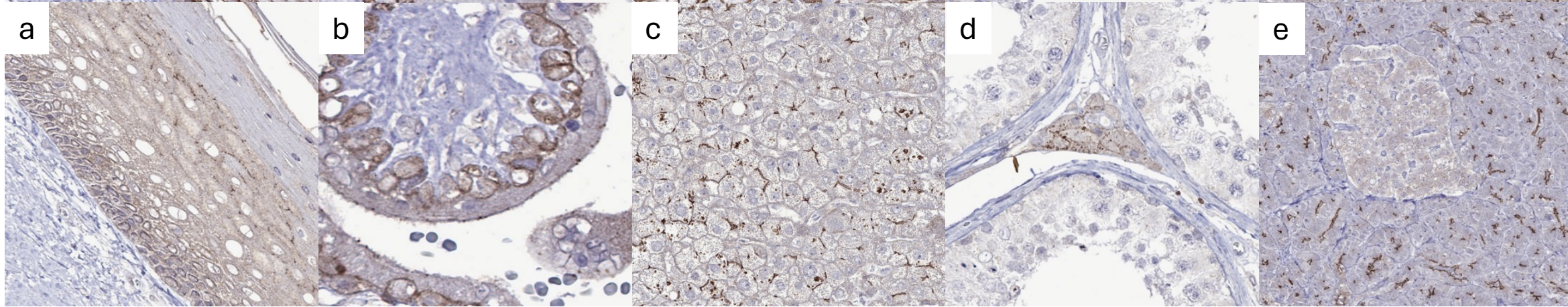

Supplement: S1 Fig — Using clone OC-3F10, a comparable, although slightly weaker, staining was seen in the ectocervix (a), the placenta (b), the liver (c), the testis (d), and the pancreas (e). The images A-E and a-e are from consecutive tissue sections. (PDF) [file pone.0321105.s001.pdf]
